# Diet-induced obesity and low testosterone increase neuroinflammation and impair neural function

**DOI:** 10.1186/s12974-014-0162-y

**Published:** 2014-09-16

**Authors:** Anusha Jayaraman, Daniella Lent-Schochet, Christian J Pike

**Affiliations:** Davis School of Gerontology, University of Southern California, 3715 McClintock Avenue, Los Angeles, CA 90089 USA

**Keywords:** Central nervous system, Diet-induced obesity, Glia, Neuroinflammation, Peripheral nervous system, Testosterone

## Abstract

**Background:**

Low testosterone and obesity are independent risk factors for dysfunction of the nervous system including neurodegenerative disorders such as Alzheimer’s disease (AD). In this study, we investigate the independent and cooperative interactions of testosterone and diet-induced obesity on metabolic, inflammatory, and neural health indices in the central and peripheral nervous systems.

**Methods:**

Male C57B6/J mice were maintained on normal or high-fat diet under varying testosterone conditions for a four-month treatment period, after which metabolic indices were measured and RNA isolated from cerebral cortex and sciatic nerve. Cortices were used to generate mixed glial cultures, upon which embryonic cerebrocortical neurons were co-cultured for assessment of neuron survival and neurite outgrowth. Peripheral nerve damage was determined using paw-withdrawal assay, myelin sheath protein expression levels, and Na^+^,K^+^-ATPase activity levels.

**Results:**

Our results demonstrate that detrimental effects on both metabolic (blood glucose, insulin sensitivity) and proinflammatory (cytokine expression) responses caused by diet-induced obesity are exacerbated by testosterone depletion. Mixed glial cultures generated from obese mice retain elevated cytokine expression, although low testosterone effects do not persist *ex vivo*. Primary neurons co-cultured with glial cultures generated from high-fat fed animals exhibit reduced survival and poorer neurite outgrowth. In addition, low testosterone and diet-induced obesity combine to increase inflammation and evidence of nerve damage in the peripheral nervous system.

**Conclusions:**

Testosterone and diet-induced obesity independently and cooperatively regulate neuroinflammation in central and peripheral nervous systems, which may contribute to observed impairments in neural health. Together, our findings suggest that low testosterone and obesity are interactive regulators of neuroinflammation that, in combination with adipose-derived inflammatory pathways and other factors, increase the risk of downstream disorders including type 2 diabetes and Alzheimer’s disease.

## Background

Normal aging is associated with a wide range of physiological changes that independently and cooperatively impact the functioning of the nervous system. One such age change is the depletion of testosterone in men. Age-related testosterone loss is linked to dysfunction and disease in several androgen-responsive tissues including adipose tissue and brain [1,2]. In brain, low testosterone is associated with significant impairment in select aspects of cognition in aging men [3,4], which rodent studies suggest could reflect the loss of testosterone regulation of behaviors [5,6], synapse formation [7], and neuron survival [8,9]. Further, low testosterone is a risk factor for Alzheimer’s disease (AD) as defined by both clinical [10–13] and neuropathological [14,15] diagnoses. In the peripheral nervous system, experimentally-induced low testosterone levels in male rats are associated with decreased expression of myelin sheath protein, which contribute to several demyelinating disorders [16]. Testosterone and its derivatives have been shown to be protective against experimental diabetic neuropathy by reversing several of the detrimental effects of low testosterone and diabetes in male rats [17]. In men with diabetes, low levels of testosterone correlate significantly with increases in neuropathy as compared to those with normal testosterone levels [18].

A second age-related change associated with poor neural outcomes is increasing adiposity. Waist circumference, body mass index, and prevalence of obesity have been shown to increase with age [19]. Obesity is a significant risk factor for development of metabolic syndrome, a collective term that includes dyslipidemia, hyperinsulinemia, and glucose intolerance [20,21]. Further, obesity is associated with inflammatory responses [22] as well as endocrine changes leading to lower testosterone levels [23]. Obesity and metabolic syndrome are also associated with increased risk for disorders including type 2 diabetes (T2D) and AD [24–27]. In the brain, high-fat diet has been shown to accelerate cognitive decline and increase insulin-resistance [28]. In the peripheral nervous system, obesity is shown to be an important factor for development of neuropathy in T2D patients [29].

Interestingly, testosterone and obesity are interactive factors that may cooperatively regulate a wide range of health measures, including nervous system function. For example, epidemiological studies have shown that men with low testosterone have higher risk of developing metabolic syndrome [30,31] and T2D [32,33]. On the other hand, central obesity and T2D reduce testosterone levels [34–37]. Moreover, testosterone therapy has been shown to reduce adiposity and T2D [38,39]. Conversely, androgen deprivation therapy for prostate cancer treatment increases the risk for metabolic syndrome and T2D [40–43].

Given the significant independent contributions of obesity and low testosterone on neural outcomes, it is important to consider the downstream effects of both these risk factors when present together. In this study, we investigate interactions between obesity and low testosterone levels on metabolic indices and neuron health in both central (CNS) and peripheral nervous system (PNS) using hormone and diet manipulations in wild-type male mice. We also examine potential contributions of inflammatory pathways in hormone and diet-induced changes in the treated animals.

## Methods

### Materials

Testosterone was purchased from Steraloids (Newport, RI, USA), solubilized in 100% ethanol, and stored at −80°C. Glucose (Life Technologies, Carlsbad, CA, USA) was dissolved in sterile water to a concentration of 0.2 g/mL. Irradiated control (10% kcal fat; Cat#D12450Bi) and high-fat (60% kcal fat; Cat#D12492i) diets were purchased from Research Diets, Inc. (New Brunswick, NJ, USA). EDTA, bovine serum albumin, NaCl, and Na_2_HPO_4_ were purchased from Thermo Fisher Scientific (Hudson, NH, USA). Imidazole was purchased from Santa Cruz Biotechnology, Inc. (Dallas, TX, USA). All other chemicals and reagents were purchased from Sigma-Aldrich (St. Louis, MO, USA).

### Animal procedures

For *in vivo* studies and primary glia cultures, male C57BL6 mice were purchased gonadectomized (GDX) and sham-GDX at 3 months of age (The Jackson Laboratory, Sacramento, CA, USA). All animals were housed individually with *ad libitum* access to food and water under a 12-h light/dark cycle. All animal procedures were conducted under a protocol that was approved by the USC Institutional Animal Care and Use Committee and in accordance with National Institute of Health standards.

For testosterone treatment, GDX male mice were implanted subcutaneously with a 30 mm length Silastic capsule (1.47 mm ID x 1.96 mm OD; Dow Corning, Midland, MI, USA) packed with dry testosterone to a length of 20 mm and capped on both ends with 5 mm of silicone glue. This capsule length has been previously demonstrated to deliver physiological levels of testosterone in male mice [44]. In a separate group of male mice, we found that this treatment yielded serum testosterone levels of 4.2 ± 0.3 ng/mL. The vehicle-treated animals were implanted with an empty capsule with the same dimensions. The diet treatments were started 1 week after GDX surgery and continued for 4 months. Prior to starting the diet treatments, base-line body weight and overnight fasting blood glucose measurements were recorded for all animals. Thereafter, body weights and food intake were measured weekly, and fasting blood glucose levels were measured every 4 weeks. Behavioral tests were conducted 1 week prior and glucose tolerance test 2 days prior to the end of the treatment period.

At the end of 4 months, mice were euthanized by CO_2_ inhalation. Brains were removed and hemisected: one-half cortex was used to generate primary glia cultures, the other half was snap-frozen on dry ice for RNA extraction and RT-PCR analyses. The sciatic nerves were dissected from both legs and snap frozen for RNA extraction, cryosectioning (for immunostaining), and for preparing lysates (for sodium potassium ATPase (Na^+^,K^+^-ATPase) assay).

### Glucose tolerance test

After overnight fasting, mice received a bolus of D-glucose (2 g/kg body weight) through oral gavage. Baseline fasting blood glucose was recorded prior to D-glucose administration and subsequent blood glucose levels were recorded 15, 30, 60, and 120 minutes after D-glucose administration. Area under the curve (AUC) was calculated using GraphPad Prism Software v5.02.

### RNA isolation and real-time PCR

For RNA extractions, cortex and sciatic nerve from each treated animal and primary glia cultures were homogenized using TRIzol reagent (Invitrogen Corporation, Carlsbad, CA, USA) and processed for total RNA extraction as per manufacturer’s protocol, as previously described [45]. Purified total RNA (1 μg) was used from each sample for reverse transcription using the iScript cDNA synthesis system (Bio-Rad, Hercules, CA, USA) and the resulting cDNA was used for real-time quantitative PCR carried out using Bio-Rad CFX Connect™ (Bio-Rad). Relative quantification of mRNA levels from various treated samples was determined by the ∆∆Ct method [46] after normalizing with the corresponding β-actin levels from samples. In addition, the PCR products were qualitatively analyzed by electrophoresis using 1% agarose gels. The following primer pairs were used: tumor necrosis factor alpha (TNFα), forward: 5′-GCCTGTAGCCCACGTCGTAG-3′, reverse: 5′-TTGGGCACATTGACCTCAGC-3′; interleukin-1β (IL-1β), forward: 5′-CCCAAGCAATACCCAAAGAA-3′, reverse: 5′-GCTTGTGCTCTGCTTGTGA-3′; P0, forward: 5′-TGTGGTTTACACGGACAGGG-3′, reverse: 5′-AGAGCAACAGCAGCAACAG-3′; β-actin, forward: 5′-AGCCATGTACGTAGCCATCC-3′, reverse: 5′-CTCTCAGCTGTGGTGGTGAA-3′.

### Primary glia cultures and neuron-glia co-cultures

Adult primary mixed glia were obtained according to previously described protocol [47] from the cortex of each individual mouse. Dissected cortices were mechanically dissociated, then plated onto poly-D-lysine coated flasks containing DMEM-F12/20% FBS and placed in a humidified incubator at 37°C with 5% CO_2_. The medium was changed every three days until the cultures were grown to confluency. Confluent cultures were re-plated onto poly-D-lysine-coated 24-well plates. The cultures were shifted to serum-free DMEM/F12 1 to 3 days prior to use in experiments. For neuron-glia co-culture studies, timed-pregnant female C57BL6 mice (Harlan Laboratories Inc., Livermore, CA, USA) were killed via CO_2_ inhalation and embryonic day 16 to 17 pups were collected for preparation of neuronal cultures. Primary cortical neurons were plated on the mixed glia at a density of 2.5 × 10^4^ cells/cm^2^ for cell viability assays, and 0.5 × 10^4^ cells/cm^2^ for neurite outgrowth studies. A parallel set of primary cortical neurons were plated at similar densities directly on poly-D-lysine coated 24-well plates and maintained in conditioned media collected from the mixed glial cultures.

### Cell viability and neurite outgrowth

For neuron viability and neurite outgrowth experiments, cells were fixed with ice-cold 4% paraformaldehyde 24 to 48 h after plating neurons. The fixed cells were immunostained with the neuron-specific marker β-tubulin III antibody (5 μg/mL; R&D Systems, Minneapolis, MN, USA) overnight at 4°C then processed with standard avidin: biotinylated enzyme complex immunocytochemistry using the Vector Elite ABC kit (Vector Laboratories, Burlingame, CA, USA); labeling was visualized by diaminobenzidine. Stained cultures were rinsed and stored in ice cold PBS until quantitation. For determining neuron survival in both neuron-glia co-cultures and neuron-only cultures, stained cells were counted in four separate fields (in a predetermined, regular pattern) per well, three wells per condition. For determining the extent of neurite outgrowth and average neurite length of neurons, 50 cells per condition were examined. The total number of neurites per cell and the average neurite length in each cell were determined using Neuron J software v.1.4.2.

### Macrophage infiltration

Twenty 40-mm length pieces of sciatic nerve from each treated animal were fixed in 4% PBS-buffered paraformaldehyde for 2 h at 4°C, rinsed with PBS, and then stored overnight in 20% sucrose solution. These tissues were then embedded in optimal temperature cutting compound and rapidly frozen. Seven-micron thick transverse-sections were cut with cryostat microtome CM1800 (Leica Microsystems Inc., Buffalo Grove, IL, USA) and mounted on microscope slides. Sections were immunostained with an antibody against the macrophage/microglia-specific Iba1 protein (1:500, Wako Chemicals, Richmond, VA, USA) overnight at 4°C and processed for immunohistochemistry as described in the previous section. Slides were rinsed, dehydrated through a graded series of alcohols, and cover-slipped with permanent mounting medium.

### Thermal nociceptive threshold

The nociceptive threshold to heat was measured using a paw withdrawal assay. A plexiglass chamber was placed over a hotplate and the temperature was maintained at 20°C. After placing the animal in the chamber, the temperature was gradually increased at the rate of 5°C/min until reaching a 50°C maximum. The threshold was measured as the temperature at which the animal shows the first sign of discomfort (i.e., paw withdrawal or licking of hind paw). For paw withdrawal latency measurements, the hotplate was maintained at 50°C and the latency was measured as the time from placement in the chamber until the animal displayed the first signs of discomfort. Animals were tested twice for each measurement with an interval of 5 min between repeats and an interval of at least 30 m in between threshold measurements and latency measurements.

### Na^+^,K^+^-ATPase assay

Na^+^,K^+^-ATPase activity was measured using sciatic nerve samples homogenized in a chilled solution of 0.25 M sucrose, 6 mM EGTA, and 10 mM Tris, at pH 7.5. Na^+^,K^+^-ATPase activity was determined colorimetrically at 700 nm using Spectramax 250 microplate reader (Molecular Devices, Sunnyvale, CA, USA) as previously described [48]. Optical density values were analyzed using SoftMax Pro 5 software. Protein content in homogenates was determined by bicinchoninic acid method (Promega) with bovine serum albumin as standard.

### Statistical analyses

Raw data were statistically analyzed using two-way ANOVA to identify simple main effects of diet and hormone status and diet X hormone interactions. Significant main effects were subsequently analyzed using Bonferroni test to compare between-group differences. Significance was indicated by *P* ≤0.05.

## Results

### Low testosterone and high-fat diet increases metabolic indices

**Figure 1:**
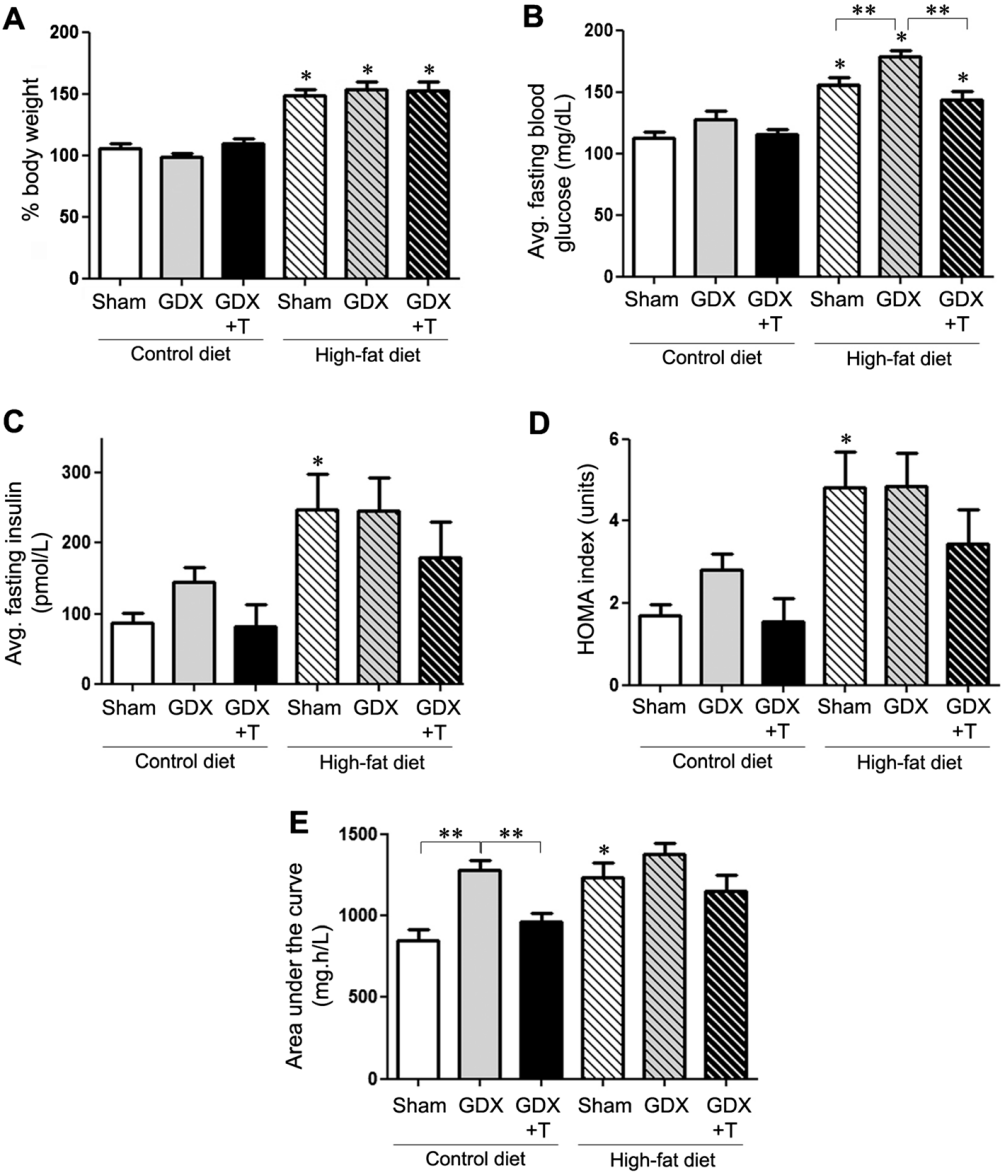
**Low testosterone and high-fat diet increases metabolic indices.** **(A)** Graphical representation of the percentage differences in average body weight of animals in sham GDX (Sham), GDX, and GDX mice with testosterone treatment (GDX + T) in both control-diet and high-fat diet. **(B, C)**. Graphical representations of average fasting blood glucose and insulin in each treated group. (**(D)** Graphical representation of HOMA index, which is representative of level of insulin resistance in each group. **(E)** Graphical representation of area under the curve (AUC) for glucose tolerance test (0 to 120 min) from each group of animals. Statistical significance is based on ANOVA followed by Bonferroni. * *P* ≤0.05 between the diet groups; ** *P* ≤0.05 between hormone groups; N ≥6.

To determine the effects of high-fat diet and low testosterone on obesity and T2D, we investigated several metabolic indices. There was a significant main effect of diet (F_1,40_ = 138.3; *P* <0.001) but not testosterone (F_2,40_ = 0.48; *P* = 0.62) in mice where high-fat diet was associated with a significant increase in body weight (Figure 1A). Fasting blood glucose levels showed significant effects for both diet (F_1,40_ = 73.0; *P* <0.01) and testosterone (F_2,40_ = 8.5; *P* = 0.001). Interestingly, GDX caused increases in fasting blood glucose in high-fat diet treatment as compared to the respective sham-GDX groups, effects that were significantly reversed by testosterone treatment. A similar non-significant trend was also seen in the control diet animals (Figure 1B). Fasting insulin levels significantly increased with diet (F_1,38_ = 16.2; *P* <0.001) and not testosterone (F_2,38_ = 1.6; *P* = 0.22), with higher fasting insulin values in the high-fat diet-fed sham-GDX as compared to the sham-GDX control diet animals. We also observed a non-significant trend of reduced fasting insulin levels by testosterone in both the diet groups (Figure 1C). Similar results were observed with HOMA index, a measure of insulin resistance based upon glucose and insulin levels where only a significant main effect of diet (F_1,38_ = 21.1; *P* <0.001) was seen between the sham groups, and testosterone treatment showed a non-significant reversal of this effect (Figure 1D). In glucose tolerance test, both diet (F_1,39_ = 14.2; *P* <0.001) and hormone (F_2,39_ = 9.7; *P* <0.001) showed significant changes. The high-fat diet sham animals showed a significant increase in AUC as compared to the control diet sham animals. Castration caused a non-significant increase in AUC that was reversed by testosterone treatment in the high-fat diet group (Figure 1E). No significant interactions between diet and hormone status simple main effects were observed in any of the metabolic measures.

### Low testosterone and high-fat diet increase proinflammatory cytokines in cortex

**Figure 2:**
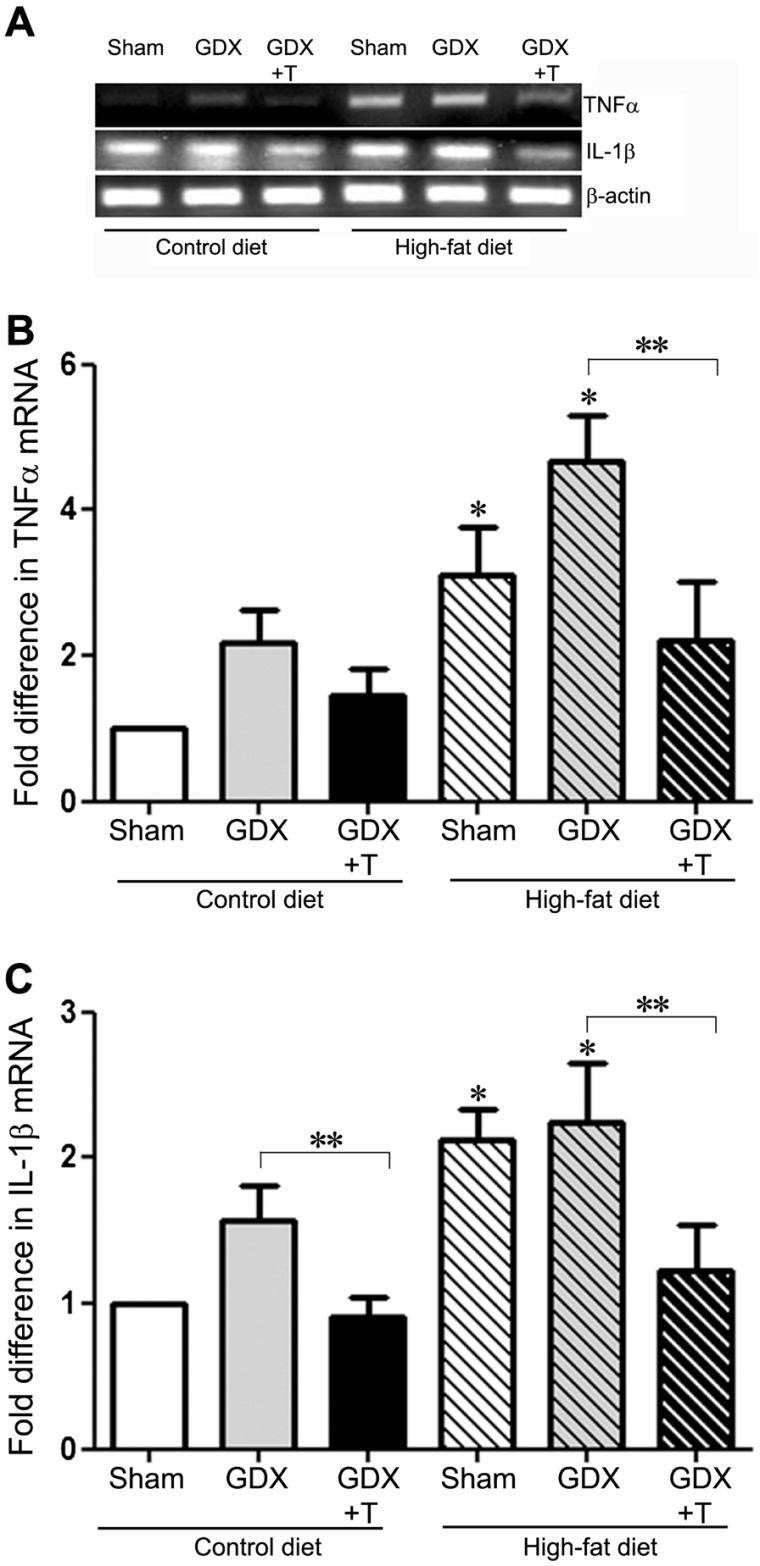
**Low testosterone and high-fat diet increase proinflammatory cytokines in cortex.** **(A)** Representative agarose gel of RT-PCR products shows the relative levels of TNFα, IL-1β, and β-actin mRNAs in sham GDX (Sham), GDX, and GDX mice with testosterone treatment (GDX + T) in both control-diet and high-fat diet. **(B)** Quantitative real-time PCR data show the mean (±SEM) expression levels compared to the Sham control group for TNFα mRNA. **(C)** Quantitative real-time PCR data show the mean (±SEM) expression levels compared to the Sham control group for IL-1β mRNA. All data are normalized with corresponding β-actin values. Statistical significance is based on ANOVA followed by Bonferroni. * *P* ≤0.05 between diet groups; ** *P* ≤0.05 between hormone groups; N ≥6.

To determine whether diet and hormone manipulations affect markers of neuroinflammation, we examined the effects of diet and hormone status on cerebrocortical mRNA levels of the proinflammatory cytokines TNFα and IL-1β across all groups. We observed a statistically significant main effect of diet on mRNA levels of TNFα (F_1,36_ = 18.2; *P* <0.001) and IL-1β (F_1,38_ = 28.2; *P* <0.001), with high-fat diet associated with elevated levels relative to matched control diet groups (Figure 2A–C). We also observed significant changes in the mRNA levels of TNFα (F_2.36_ = 5.8; *P* <0.01) and IL-1β (F_2,38_ = 12; *P* <0.001) due to hormone status, with GDX causing elevated levels in both diet groups, which was reversed by testosterone treatment (Figure 2A–C). There was no significant interaction between diet and hormone simple main effects for TNFα levels. However, a significant interaction was observed for IL-1β mRNA levels (F_2,38_ = 3.5; *P* = 0.04).

### Low testosterone and high-fat diet increase proinflammatory cytokines in primary glia cultures

**Figure 3:**
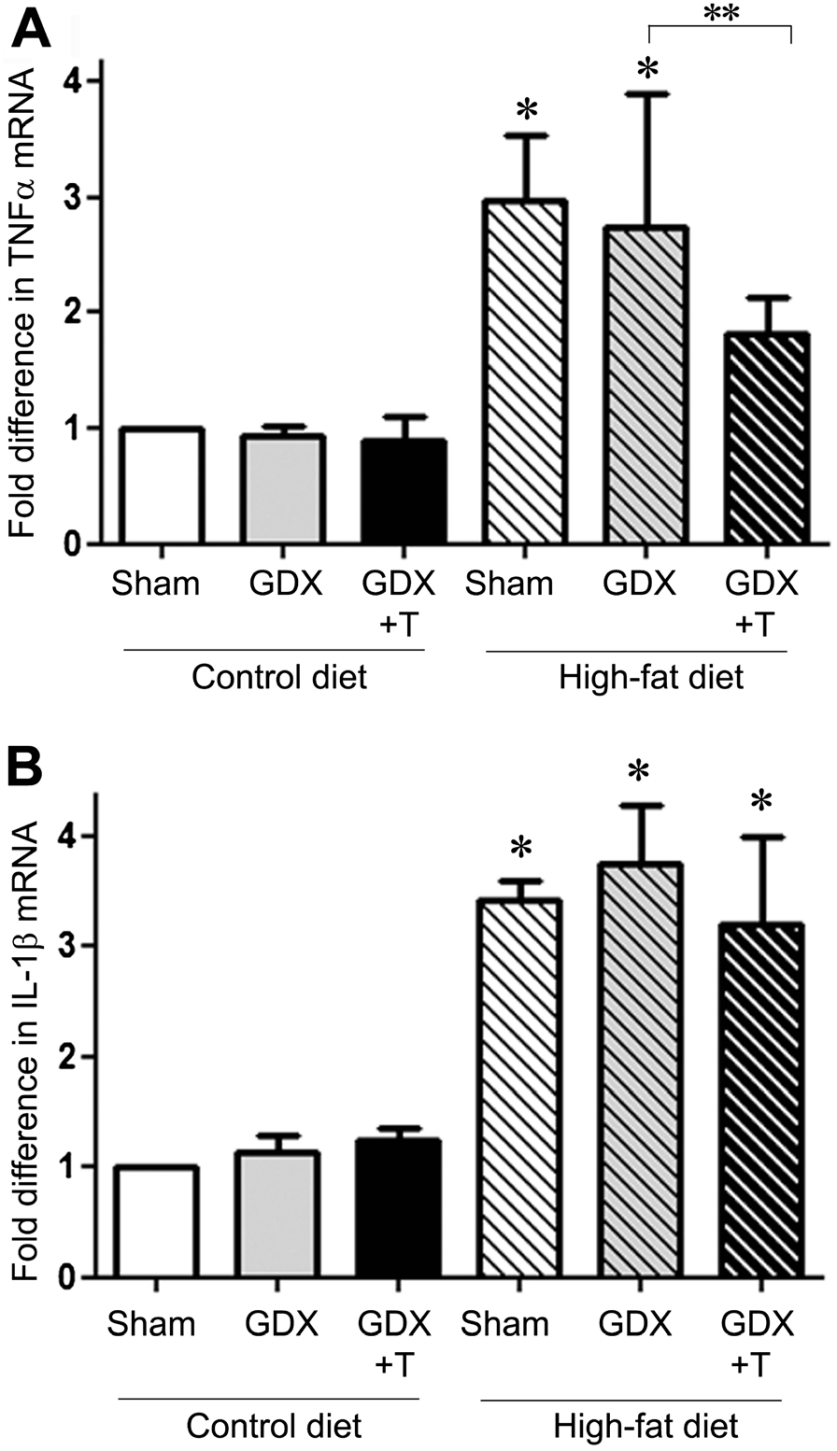
**High-fat diet increase proinflammatory cytokines in primary glial culture.** **(A)** Quantitative real-time PCR data show the mean (±SEM) expression levels compared to the Sham control group for TNFα mRNA. **(B)** Quantitative real-time PCR data show the mean (±SEM) expression levels compared to the Sham control group for IL-1β mRNA. All data are normalized with corresponding β-actin values. Statistical significance is based on ANOVA followed by Bonferroni. * *P* ≤0.05 between diet groups; ** *P* ≤0.05 between hormone groups; N ≥6.

To determine whether diet and hormone manipulations have an effect on neuroinflammation in mixed glial cultures, we examined the effects of diet and hormone changes on mRNA levels of proinflammatory cytokines TNFα and IL-1β in the primary glial cultures prepared from the cortices of the treated animals. We observed statistically significant main effects of only diet (F_1,12_ = 33.4; *P* <0.001) and not hormone (F_2,12_ = 2.1; *P* = 0.17) on mRNA levels of TNFα in comparing the levels in the glial cultures from both diet groups. However, there was a significant decrease in TNFα mRNA levels with testosterone treatment in the high-fat animals (*P* <0.05) (Figure 3A). IL-1β mRNA levels were also significantly affected by diet (F_1,12_ = 49.5; *P* <0.001) but not hormones (F_2,12_ = 0.2; *P* = 0.82) (Figure 3B). Moreover, no significant interactions were observed between diet and hormone status simple main effects for either TNFα or IL-1β mRNA levels.

### High-fat diet alters glia-mediated neuron survival

**Figure 4:**
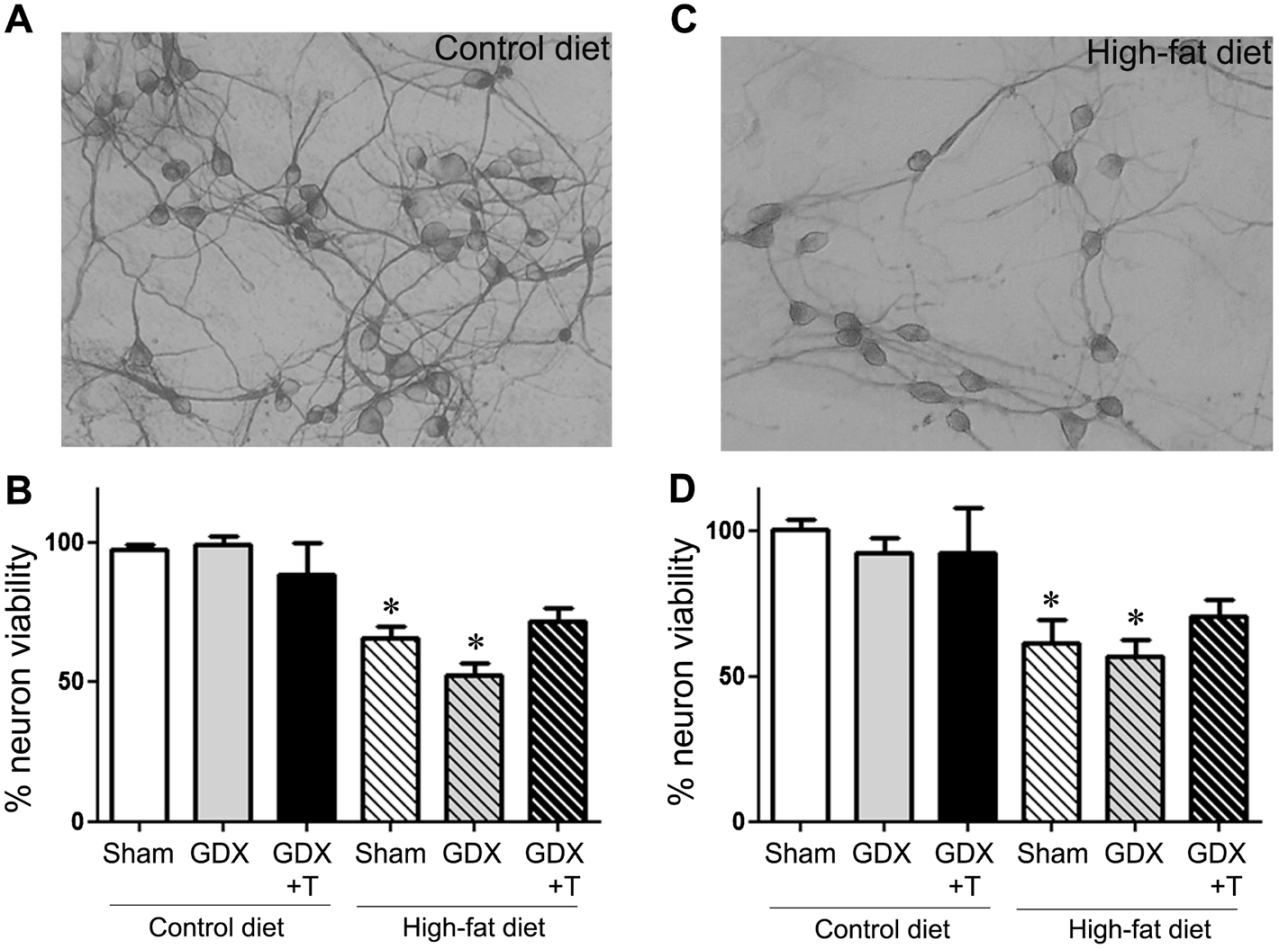
**High-fat diet derived glia cultures support reduced neuron viability.** Primary cortical neurons were plated either by themselves or on confluent primary glial cultures derived from the cortices of sham GDX (Sham), GDX, and GDX mice with testosterone treatment (GDX + T) in both control-diet and high-fat diet. Top panels show representative pictures of neurons co-cultured with control diet **(A)** and high-fat diet **(C)** derived glia. **(B)** Quantitative graph for percentage of neurons per treatment group in the neuron-glia co-culture. **(D)** Quantitative graph for percent neuron viability in conditioned glial media from each group. Data show mean cell viability (±SEM) of a representative experiment. * *P* ≤0.05 between diet groups; ** *P* ≤0.05 between hormone groups; N = 3.

We investigated whether the changes in the level of inflammatory markers observed in the glial cultures derived from cortices of animals under the two diets affected neuron survival differently. Primary cortical neurons from E17 mouse pups were co-cultured with mixed glia cultured from treated animals. We observed a significant main effect of diet alone on neuron viability in the neuron-glia co-cultures (F_1,12_ = 43.4; *P* <0.001) (Figure 4A–C). Interestingly, primary neurons growing in the conditioned media from these high-fat derived glia also showed lower viability as compared to those growing in the conditioned media from control-diet derived glia (F_1,12_ = 22.7; *P* <0.001) (Figure 4D). In both co-culture and conditioned media experiments, there were no significant changes seen due to hormone status (Figure 4C,D). No interaction between diet and hormone status simple main effects was seen either in the co-cultures or with conditioned media.

### High-fat diet alters neurite outgrowth number and length

**Figure 5:**
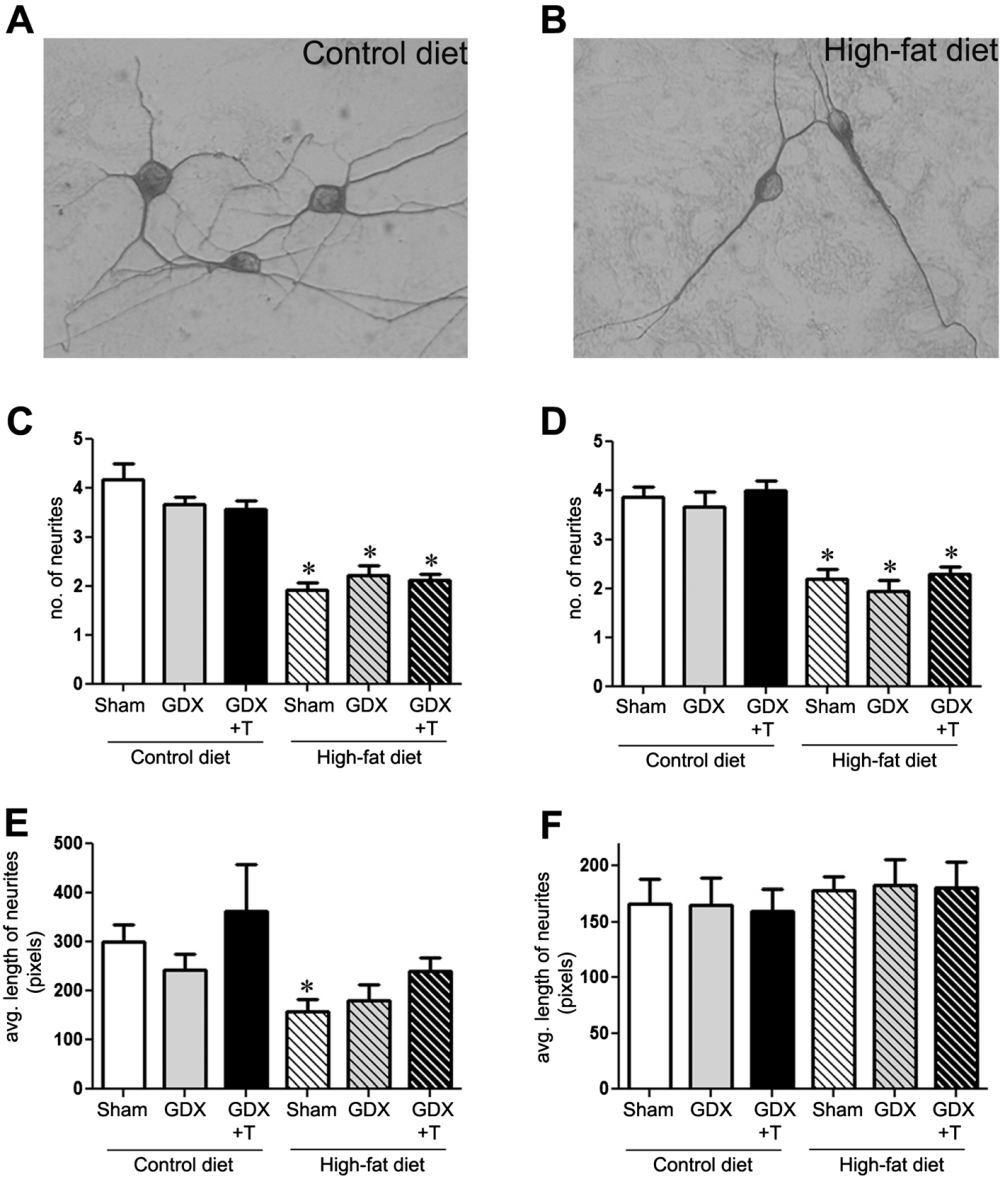
**High-fat diet-derived glia cultures yield reduced neurite outgrowths.** Primary cortical neurons were plated either by themselves or on confluent primary glial cultures derived from the cortices of sham GDX (Sham), GDX, and GDX mice with testosterone treatment (GDX + T) in both control-diet and high-fat diet. Top panels show representative pictures of neurite outgrowths on neurons co-cultured with **(A)** control diet- and **(B)** high-fat diet-derived glia. Quantitative graphs show mean **(C)** number of neurites and **(E)** length of neurites per neuron across treatment groups in the neuron-glia co-cultures. Quantitative graphs show mean **(D)** number of neurites and **(F)** length of neurites per neuron in conditioned glial media from each group. Data show mean values (±SEM) of a representative experiment. * *P* ≤0.05 between diet groups; ** *P* ≤0.05 between hormone groups; N = 3.

To determine whether high-fat diet affects neurite number and neurite length, neurons were examined under co-culture and conditioned media paradigms. We observed that neurons growing on high-fat diet derived glia (F_1,18_ = 121.2; *P* <0.001) or the conditioned media obtained from them (F_1,18_ = 102.5; *P* <0.001) had fewer numbers of neurites as compared to those growing on control-diet derived glia/conditioned media (Figure 5A–D). Moreover, the average length of neurites was significantly shorter in neurons growing on high-fat diet-derived glia (F_1,27_ = 7.6; *P* <0.05) (Figure 5E). Interestingly, there were no differences in average neurite lengths in neurons growing in either of the conditioned media (Figure 5F). As in the case of neuron survival, hormone status of the animals from which the glia were derived did not have an effect on neurite numbers and lengths (Figure 5C–F). No significant interaction was observed in neurite numbers or average neurite lengths in co-cultures and conditioned media.

### Low testosterone and high-fat diet increase macrophage infiltration in the sciatic nerve

**Figure 6:**
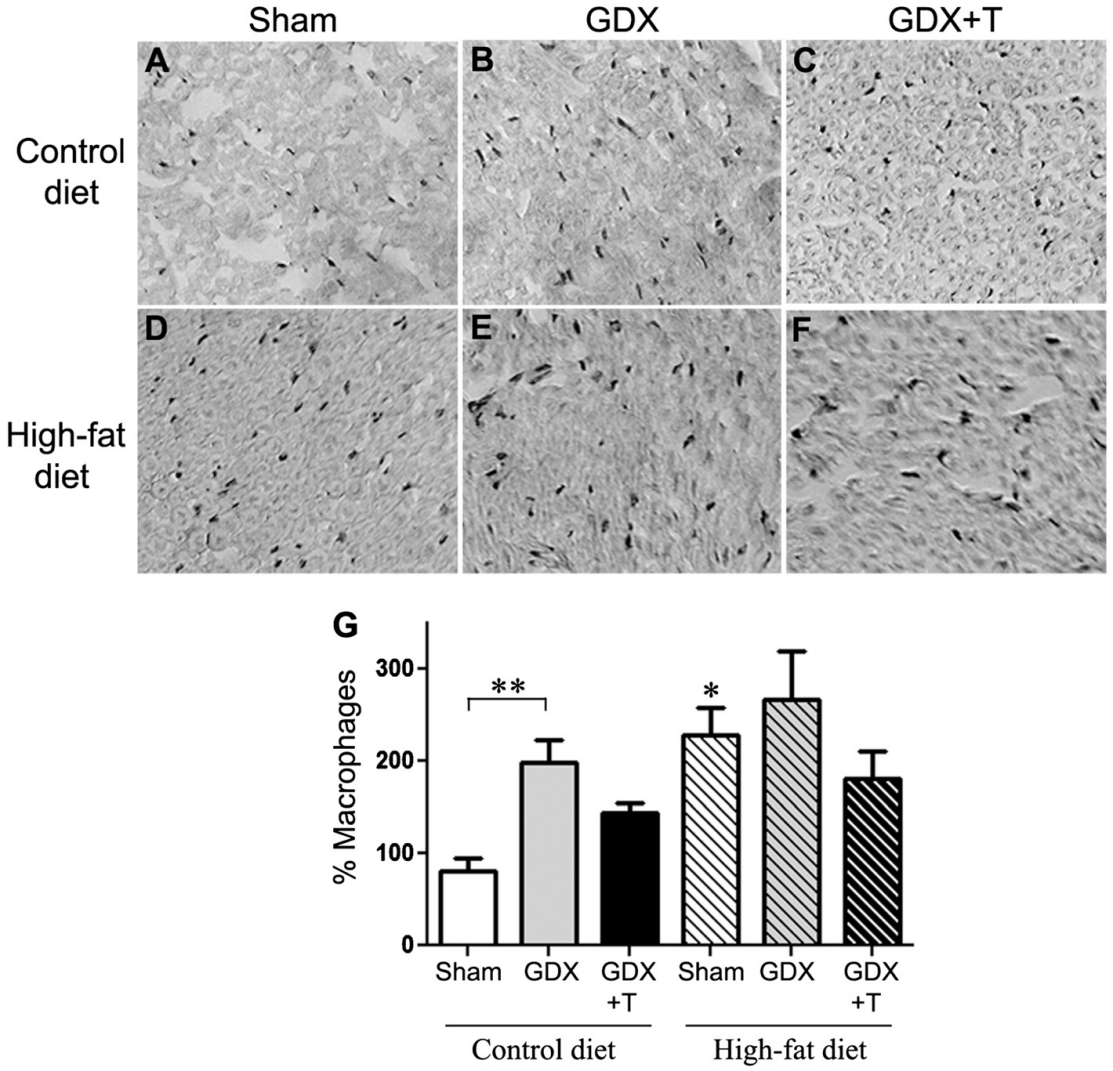
**Low testosterone and high-fat diet increase macrophage infiltration in sciatic nerve.** **(A–F)** Top panels are the representative pictures showing amount of macrophage infiltration in sciatic nerve sections in each treatment condition. **(G)** Quantitative graph showing the percentage of macrophages present in the sciatic nerve sections from each treatment group. * *P* ≤0.05 between diet groups; ** *P* ≤0.05 between hormone groups; N ≥6.

To investigate the effect of diet and hormone manipulations in the PNS, sciatic nerve sections from treated animals were examined for macrophage infiltration, which is one of the key signs of peripheral inflammatory response. We observed significant main effects of both diet (F_1,19_ = 11.6; *P* <0.01) and hormone status (F_2,19_ = 4.8; *P* <0.05) on macrophage infiltration in the sciatic nerve, with high-fat diet and GDX independently increasing the number of macrophages in the sciatic nerve sections (Figure 6A,B,D,E,G). Testosterone treatment in both the diet groups reversed the macrophage levels although non-significantly (Figure 6C,F,G). The interaction between diet and hormone status simple main effects was also not significant.

### Low testosterone and high-fat diet increase proinflammatory cytokines and decrease myelin sheath marker in sciatic nerve

**Figure 7:**
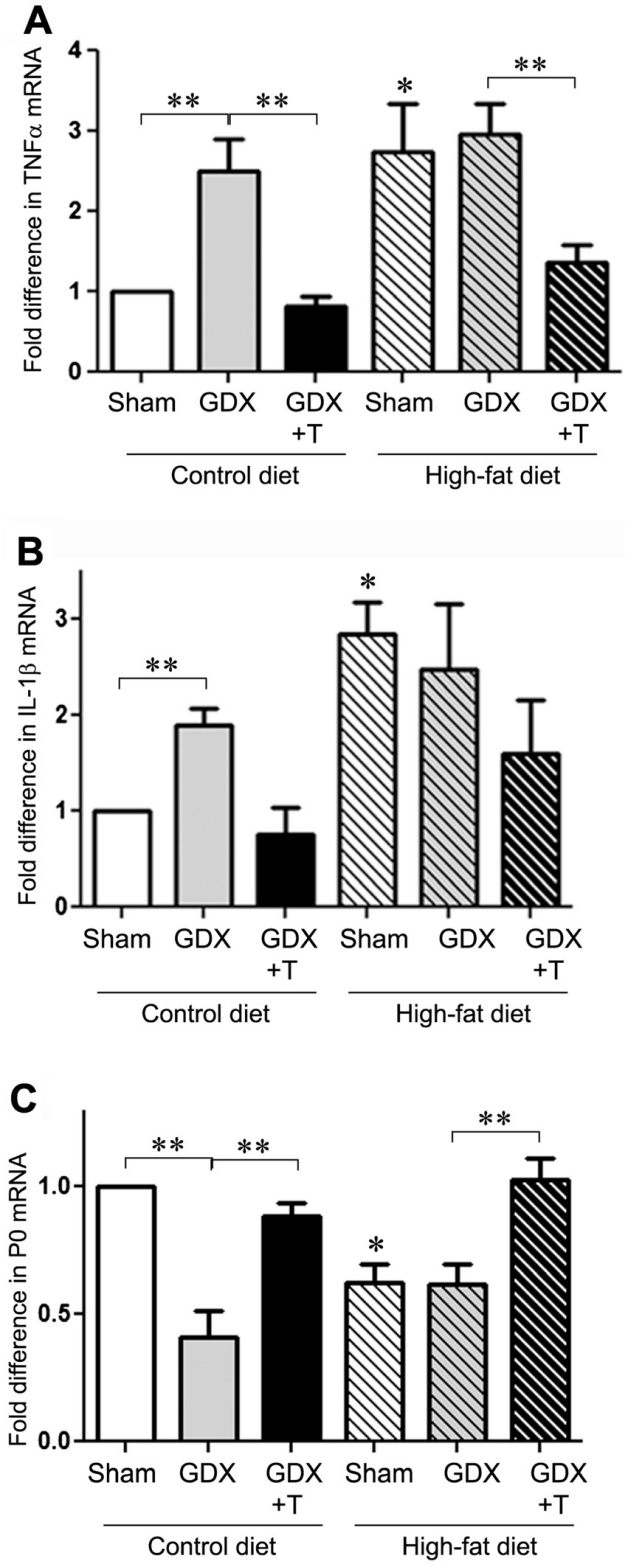
**Low testosterone and high-fat diet increase proinflammatory cytokines TNFα and IL-1b mRNA and decrease myelin sheath marker P0 mRNA in sciatic nerve.** **(A)** Quantitative real-time PCR data show the mean (±SEM) expression levels compared to the Sham control group for TNFα mRNA. **(B)** Quantitative real-time PCR data show the mean (±SEM) expression levels compared to the Sham control group for IL-1β mRNA. **(C)** Quantitative real-time PCR data show the mean (±SEM) expression levels compared to the Sham control group for P0 mRNA. All data are normalized with corresponding β-actin values. Statistical significance is based on ANOVA followed by Bonferroni. * *P* ≤0.05 between diet groups; ** *P* ≤0.05 between hormone groups; N ≥6.

To determine whether the low testosterone levels and high-fat diet alter proinflammatory cytokines in the PNS as they did in the cortex, mRNA levels of TNFα and IL-1β were observed in the sciatic nerve samples. Our results showed a significant increase in TNFα mRNA expression by both diet (F_1,30_ = 13.4; *P* = 0.001) and hormone (F_2,30_ = 12.4; *P* <0.001), which was reversed by testosterone treatment in both the diet-fed animals (*P* <0.05) (Figure 7A,B). Similarly, IL-1β mRNA was significantly affected by diet (F_1,12_ = 12.5; *P* <0.05) and hormone (F_2,12_ = 4.2; *P* <0.05). Testosterone treatment decreased IL-1β mRNA expression in both diet groups, with significant effects in the control-diet animals (*P* <0.001). In addition, we investigated the effect of low testosterone and high-fat diet on myelin sheath protein (P0) in the sciatic nerve, which is shown to decrease in case of diabetic neuropathy [49]. We observed that P0 mRNA levels significantly changed with hormone status (F_2,18_ = 18.5; *P* <0.001), with GDX decreasing the P0 mRNA levels, which was restored with testosterone treatment in both the diet groups (Figure 7C). We also observed a decrease in P0 mRNA in high-fat diet sham animals as compared to the control diet sham animals (*P* <0.01). There was significant interaction observed between diet and hormone status for TNFα mRNA levels (F_2,30_ = 3.3; *P* = 0.05) and P0 mRNA levels (F_2,18_ = 9.4; *P* = 0.0016). However, no such interaction was seen for IL-1β mRNA expression.

### Low testosterone and high-fat diet promote hyperalgesia and Na^+^,K^+^-ATPase activity in sciatic nerve

**Figure 8:**
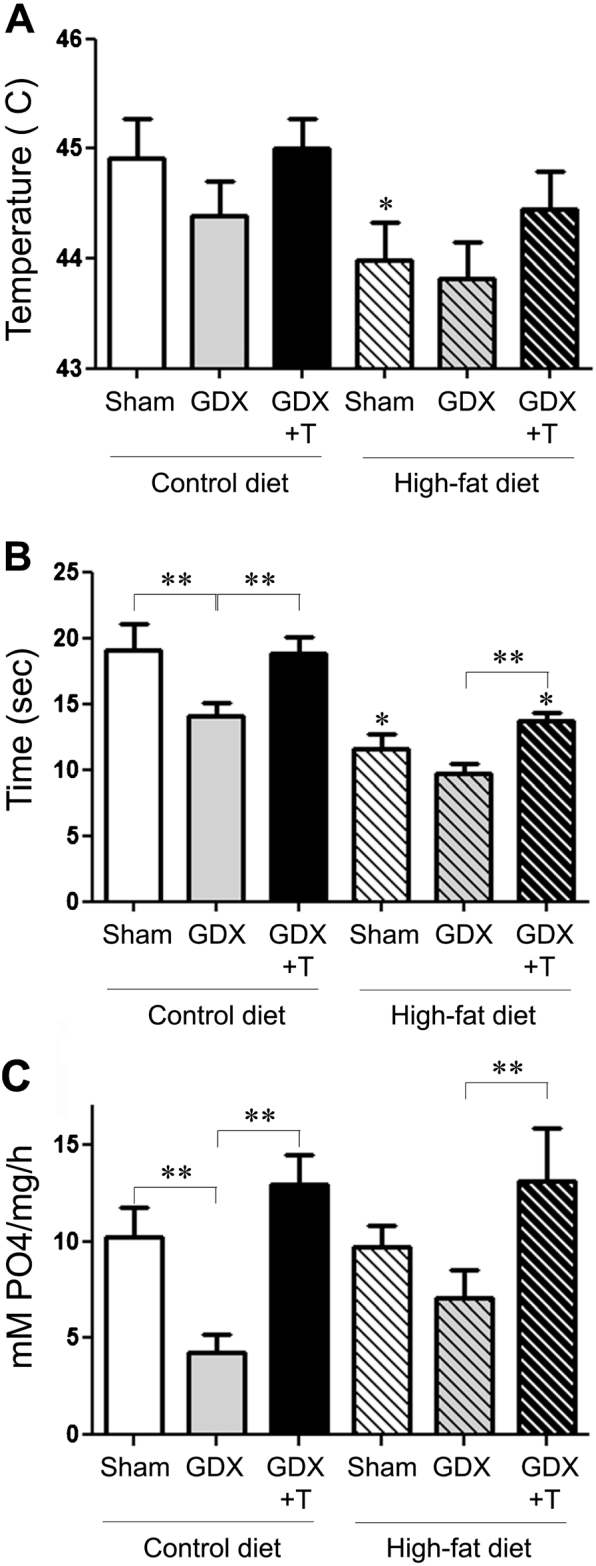
**Low testosterone and high-fat diet promote hyperalgesia and Na** ^**+**^ **,K** ^**+**^ **-ATPase activity in sciatic nerve.** **(A)** Quantitative graph shows the mean (±SEM) temperature threshold for sham GDX (Sham), GDX, and GDX mice with testosterone treatment (GDX + T) in both control-diet and high-fat diet fed mice. **(B)** Quantitative graph show the mean (±SEM) latency to thermal nociception (at 50°C) in different animal groups. **(C)** Quantitative graph shows the mean (±SEM) activity levels of Na^+^,K^+^-ATPase in the sciatic nerve of animals in the different treatment groups. Statistical significance is based on ANOVA followed by Bonferroni. * *P* ≤0.05 between diet groups; ** *P* ≤0.05 between hormone groups; N ≥6.

To investigate whether low testosterone and high-fat diet promote other markers of peripheral nerve damage, we did a hot plate paw withdrawal assay one week prior to the end of the treatment period. We observed that diet (F_1,38_ = 6.2; *P* <0.05), but not hormone (F_2,38_ = 1.8; *P* = 0.18), induced significant changes in the threshold to heat between different animal groups. Interestingly, hyperalgesia was induced in the respective groups of animals as observed by significant main effects of both diet (F_1,38_ = 28.9; *P* <0.001) and hormone (F_2,38_ = 6.3; *P* <0.005) on latency to heat in these animals (Figure 8A, B). Testosterone treatment was able to reverse these in both diet conditions (Figure 8A,B). We also looked at the Na^+^,K^+^-ATPase activity in the sciatic nerve samples from the different animal groups. We observed that hormone status altered the Na^+^,K^+^-ATPase activity levels (F_2,32_ = 10.1; *P* <0.001), with GDX lowering the enzyme activity levels in both diet conditions which was rescued by testosterone treatment (Figure 8C). Diet and hormone status together had no interactive effect on threshold and latency to heat as well as Na^+^,K^+^-ATPase activity.

## Discussion

Prior work has identified obesity and low testosterone as risk factors for the development of the metabolic syndrome [50–53] and T2D [54–57]. Interestingly, both obesity and low testosterone are also risk factors for neural dysfunction, including cognitive impairment [58–61] and development of AD [10,11,14]. Levels of obesity and testosterone are often inversely correlated, suggesting the possibility that they are interactive factors [62]. In this study, we investigated the individual and combined effects of obesity and testosterone status on neural outcomes. Our results demonstrate that diet-induced obesity causes significant metabolic disturbances and impairs central and peripheral nervous systems. Testosterone status also affected metabolic and neural measures. In general, obesity-related changes were worsened by low testosterone and improved by testosterone treatment; however, this relationship was not statistically significant in several instances. Further, our data suggest that a common pathway that may contribute to obesity and testosterone effects is regulation of inflammation.

We observed that metabolic measures were affected by both diet-induced obesity and testosterone status. In some cases, fasting blood glucose levels were independently and additively increased by GDX-induced testosterone depletion and high-fat diet. Importantly, testosterone treatment significantly reduced fasting glucose under both the normal and high-fat diets, demonstrating potential therapeutic efficacy of testosterone supplementation. For measures of fasting insulin, insulin resistance (HOMA index), and glucose tolerance, low testosterone tended to exacerbate and or testosterone treatment improved outcomes. Because testosterone status did not significantly affect body weight, testosterone’s effects likely do not indicate an indirect result on adiposity but rather regulatory action(s) on other aspects of metabolic homeostasis. Prior work in rodents has shown diet-induced obesity induces insulin resistance in rat brain [63] and that testosterone replacement improves insulin sensitivity in obese rats [64]. Our findings are consistent with the human literature, which indicates that (i) testosterone levels are inversely correlated to insulin resistance and T2D in healthy [30,65] as well as obese men [66], and (ii) androgen therapy can improve some metabolic measures in overweight men with low testosterone [67–69].

In addition to impairing metabolic function, both obesity and low testosterone are linked with promotion of inflammatory pathways [70–72] and exert harmful actions on the central [73–75] and peripheral [29,76] nervous systems. To investigate these relationships and their potential interactions, we first examined the effects of experimentally-induced low testosterone and obesity on brain levels of two established pro-inflammatory markers, TNFα [77] and IL-1β [78]. Our data demonstrate that low testosterone and obesity independently increased cerebrocortical mRNA levels of both TNFα and IL-1β. Although there was not a statistically significant additive effect of the two factors on cytokine expression, testosterone treatment significantly lowered TNFα and IL-1β expression to near basal levels even in obese mice, indicating a protective benefit of testosterone across diet conditions. Similar to the observations on metabolic outcomes, our findings on cytokine expression are consistent with both individual and inter-related effects of testosterone and obesity. Because many beneficial effects of testosterone, including inhibition of proinflammatory cytokine expression [79] and neuroprotection [80,81], are dependent upon androgen receptors, the observed effects of testosterone in this study may involve androgen receptor activation. However, testosterone can be converted by the enzyme aromatase into estradiol, which is also known to exert anti-inflammatory [82] and neuroprotective [83] actions. Additional research will be needed to elucidate the relative contributions of androgen and estrogen pathways to the observed relationships between testosterone, obesity, inflammation, and neural outcomes.

Because glia are the primary sources of proinflammatory molecules in the CNS, we considered whether glia may contribute to the established neural effects of low testosterone and obesity. We generated mixed glial cultures from treated mice, an established paradigm in which glial phenotype established *in vivo* is retained in culture [47,84,85], then plated upon them naïve embryonic cerebrocortical neurons. An advantage of this approach is that it allows isolated assessment of glial effects on neuronal health without influence of systemic alterations associated with obesity (e.g*.*, alterations in glucose and insulin signaling). We observed significantly poorer survival of neurons grown on glia from mice maintained on high-fat diet as well as significant reductions in the numbers and lengths of neurites. Since testosterone can affect glial function [86] and improve neuronal growth and survival [87–89], it was unexpected that testosterone status exhibited rather modest effects on neural health indices with the only significant response being an increase in survival in the testosterone-treated, high-fat diet group. Note that testosterone status was controlled *in vivo* but not *in vitro*, suggesting the possibility that its effects on glia and neurons require maintained exposure to the hormone.

Importantly, the inhibitory effects of cultured glia on neurons were largely reproduced by exposing neuron cultures to media conditioned by glial cultures generated from mice maintained on a high-fat diet. Both neuron survival and neurite number were reduced to similar levels in neurons either co-cultured with glia from fat-fed mice or conditioned media from the fat-fed glial cultures. This finding suggests that soluble factors, possibly including toxic proinflammatory cytokines secreted by astrocytes and/or microglia, adversely affected neuronal viability. Consistent with this possibility, we observed significantly increased expression of TNFα and IL-1β in glia cultures derived from obese mice. In prior work, it has been shown that TNFα has inhibitory effects on neuron survival, differentiation, and neurite outgrowth [90–92]. Similarly, IL-1β treatment has been shown to induce synapse loss and inhibit differentiation of neurons [93,94]. Interestingly, mean neurite length was not reduced by the conditioned media, indicating a significant contribution of glial cell surface components in the regulation of neurite length.

We also considered the possible effects of low testosterone and obesity on health and functioning of the PNS. A major PNS-related complication of T2D is diabetic neuropathy. Peripheral diabetic neuropathy involves several changes, including increased induction of inflammatory cytokines, macrophage infiltration, decreased expression of P0, thermal nociception, and biochemical changes in the nerves [95]. In our diet-induced pre-diabetes model, we investigated some of these changes in the sciatic nerves of treated animals. Testosterone status and diet-induced obesity were associated with significant regulation of macrophage infiltration, mRNA levels of IL-1β, TNFα, and P0, and activity levels of Na^+^,K^+^-ATPase. For example, GDX-induced low testosterone and diet-induced obesity each independently decreased the expression levels of P0. Although there was no an additive effect of these factors, testosterone treatment in both control and high-fat diet groups significantly increased P0 expression to values observed in the sham-GDX control-diet group, demonstrating protection effect of testosterone across conditions. Similarly, testosterone reduced IL-1β and TNFα levels and improved the activity of Na^+^,K^+^-ATPase that was reduced by both GDX and high-fat conditions. These findings are in accordance with previous studies that have correlated the decreased expression of P0 and Na^+^,K^+^-ATPase activity to the onset of peripheral neuropathy in several animal models of diabetes [17,96] and in persons with T2D [97,98].

One clinical feature of peripheral neuropathy that is also observed in many animal models of diabetes is hyperalgesia [99,100]. For this functional endpoint, we observed threshold hyperalgesia in GDX animals that was exacerbated by high-fat diet. Importantly, testosterone prevented and/or restored thermal nociception in both diet groups. The underlying mechanism for changes in thermal nociception has been linked to inflammatory pathways. For example, up-regulation of TNFα in both CNS and PNS results in development of hyperalgesia [101,102]. Similarly, IL-1β has also been shown to induce pain hypersensitivity by activating nociceptors [103]. Our observations of IL-1β and TNFα changing in parallel with thermal nociception are consistent with prior observations of a mechanistic link between inflammation and hyperalgesia.

## Conclusions

Our study provides novel insights into the individual and interactive effects of low testosterone- and diet-induced obesity on nervous system function. The most significant observation is that the combination of low testosterone and obesity worsen several metabolic and inflammatory indices, which in turn are largely reversed by testosterone treatment. The inverse relationship between various endpoints and proinflammatory cytokine levels suggests a possible mechanism by which obesity and testosterone levels may affect the health of both CNS and PNS. Low testosterone and obesity are commonly present together in many middle-age and aged men. Our findings suggest that these factors have individual and combined effects. Continued investigation of the interplay between these two factors in the nervous system would be beneficial not only in increasing our understanding of their long-term impact, but also in designing and optimizing potential hormone-based approaches as a therapeutic strategy to overcome some of their negative outcomes.

## Abbreviations

AD: Alzheimer’s disease
AUC: Area under the curve
CNS: Central nervous system
GDX: Gonadectomized
IL-1β: Interleukin-1β
Na^+^,K^+^-ATPase: Sodium potassium ATPase
P0: Myelin protein 0
PNS: Peripheral nervous system
T2D: Type 2 diabetes
TNFα: Tumor necrosis factor α
